# Molecular and Phytochemical Characteristics of Flower Color and Scent Compounds in Dog Rose (*Rosa canina* L.)

**DOI:** 10.3390/molecules29133145

**Published:** 2024-07-02

**Authors:** Parisa Jariani, Ali-Akbar Shahnejat-Bushehri, Roohangiz Naderi, Meisam Zargar, Mohammad Reza Naghavi

**Affiliations:** 1 Division of Biotechnology, Department of Agronomy and Plant Breeding, College of Agricultural and Natural Resources, University of Tehran, Karaj 31587-77871, Iran; parisa.jariani@ut.ac.ir (P.J.); ashah@ut.ac.ir (A.-A.S.-B.); 2 Department of Horticulture Science, College of Agriculture and Natural Resources, University of Tehran, Karaj 31587-77871, Iran; rnaderi@ut.ac.ir; 3 Department of Agrobiotechnology, Institute of Agriculture, RUDN University, 117198 Moscow, Russia; zargar-m@rudn.ru

**Keywords:** aroma, essential oils, flavonoids, *LC-ESI-MS*, qRT-PCR, *Rosa canina*

## Abstract

This study delves into the chemical and genetic determinants of petal color and fragrance in *Rosa canina* L., a wild rose species prized for its pharmacological and cosmetic uses. Comparative analysis of white and dark pink *R. canina* flowers revealed that the former harbors significantly higher levels of total phenolics (TPC) and flavonoids (TFC), while the latter is distinguished by elevated total anthocyanins (TAC). Essential oils in the petals were predominantly composed of aliphatic hydrocarbons, with phenolic content chiefly constituted by flavonols and anthocyanins. Notably, gene expression analysis showed an upregulation in most genes associated with petal color and scent biosynthesis in white buds compared to dark pink open flowers. However, anthocyanin synthase (*ANS*) and its regulatory gene *RhMYB1* exhibited comparable expression levels across both flower hues. LC-MS profiling identified Rutin, kaempferol, quercetin, and their derivatives as key flavonoid constituents, alongside cyanidin and delphinidin as the primary anthocyanin compounds. The findings suggest a potential feedback inhibition of anthocyanin biosynthesis in white flowers. These insights pave the way for the targeted enhancement of *R. canina* floral traits through metabolic and genetic engineering strategies.

## 1. Introduction

Rose (*Rosa* L.) is a well-known ornamental plant that is admired for its attractive and aromatic petals [1]. The petals exhibit a wide range of colors and scents, which are of great interest for several industries, especially cosmetics and perfumery. The petals are the main source of rose oils, also known as “liquid gold”, which are highly sought-after for their distinctive fragrance and therapeutic benefits. However, rose oils are rare and costly, as roses have a low oil yield and no adequate natural or synthetic alternatives [2,3,4]. Hence, genetic diversity is essential for enhancing the quality and quantity of rose oils through breeding programs [5]. *R. canina*, or the dog rose, is a variable and climbing wild rose species that is native to Europe, northwest Africa, and western Asia [6]. It belongs to the Caninae section of the Rosa genus, which consists of about 20 species that share common features such as prickles, pinnate leaves, and deciduous sepals [7,8]. *R. canina* has a wide distribution in Iran, which is regarded as its primary center of diversity [9]. It produces flowers with white to pink petals that differ in color and shape among various populations. The flowers are followed by bright red fruits, called hips, that contain numerous seeds and are rich in vitamin C and other bioactive compounds [10,11,12]. *R. canina* has a long history of use for medicinal, cosmetic, and culinary purposes [13]. In particular, it is a valuable source of rose oils, which are obtained from the petals and used in perfumery and aromatherapy for their unique scent and therapeutic properties.

The emission of volatile organic compounds (VOCs) and the accumulation of various pigments, such as flavonoids and anthocyanins, are important functions of petals [14]. VOCs are produced by flowers through three main metabolic pathways: terpenoids, phenylpropanoids/benzenoids, and fatty acid derivatives [4]. These pathways utilize common precursors from the shikimate (SA), methylerythritol 4-phosphate (MEP), and mevalonate (MVA) pathways [14,15]. Terpenes, a major class of VOCs, are synthesized either in plastids via the MEP pathway or in cytosol via the MVA pathway [16]. Phenylpropanoids generate aromatic compounds, such as benzyl acetate, eugenol, methyl benzoate, phenylethyl acetate, and phenylethanol, using L-phenylalanine from the SA pathway as the main precursor [17]. Fatty acid derivatives, another class of VOCs, comprise low-molecular weight alcohols, aldehydes, and lipids. They are catalyzed by the lipoxygenase (LOX) pathway using linolenic and linoleic acid as initiators [18]. The color of rose flowers is mainly determined by flavonoids, anthocyanins, carotenoids, betalains, and other floral pigments, with flavonoids being the predominant group of secondary metabolites in plants [19]. Phenolic compounds are significant in *R. canina* due to their complex structure and antioxidant activity [20]. Flavonoids are the principal phenolic compounds in plants and have various biological effects [21]. Therefore, it is essential to identify and analyze different phenolic compounds in *R. canina* petals by LC-MS analysis.

Polyphenols are widely used in health and industry, but their isolation and characterization are difficult. Flavonoids are a group of polyphenolic pigments that are synthesized in the cytosol via the SA pathway [22]. They originate from phenylalanine, which competes with chalcone for anthocyanin production [23,24]. Flavonoids can be divided into several subclasses, such as chalcone, aurone, flavone, flavanone, flavonol, isoflavone, catechin, anthocyanidin, and anthocyanin [25]. Anthocyanins are the main water-soluble pigments that accumulate in the vacuolar part of epidermal cells in reproductive and vegetative tissues [26]. There are six major classes of anthocyanin pigments: delphinidin, cyanidin, pelargonidin, peonidin, petunidin, and malvidin. Among them, the pelargonidin and cyanidin classes are responsible for the pink, orange, and red colors of petals and belong to the main phenolic compounds [25,27,28]. Flavones and flavonols act as co-pigments with anthocyanins, enhancing the intensity of anthocyanins [26]. Molecular biology has become more relevant in the evaluation of aromatic plants in recent years [3].

Recent studies on the *Rosa* genus have unveiled a wealth of information on the complex phenolic profiles and flavonoid compounds within these species, as highlighted by Fetni et al. [29] and Behnamnia et al. [30]. These insights point to the significant potential of these constituents in promoting human health and advancing plant cultivars. However, there remains a gap in the literature regarding the molecular and chemical characteristics of dog rose petals, especially in relation to their role in floral scent and color biosynthesis. The current research seeks to fill this void by undertaking a comprehensive analysis of the essential oil composition of dog rose flowers at different developmental stages using gas chromatography–mass spectrometry (GC-MS), quantifying the total phenol and flavonoid content in petals through hydro-methanolic extracts, examining gene expression involved in flavonoid biosynthesis via qRT-PCR [31], and identifying phenolic compounds using LC-MS. The objective is to elucidate the genetic and metabolic regulation of flower color and fragrance in dog rose, which will inform strategies to enhance its scent, color, and phytochemical profiles for sustainable cultivation and production. This research not only contributes to the ongoing scientific dialogue but also aims to tap into the therapeutic and industrial potential of the *Rosa* genus.

## 2. Results

### 2.1. Chemical Compositions of the R. canina Essential Oil by GC-MS Analysis

**Figure 1:**
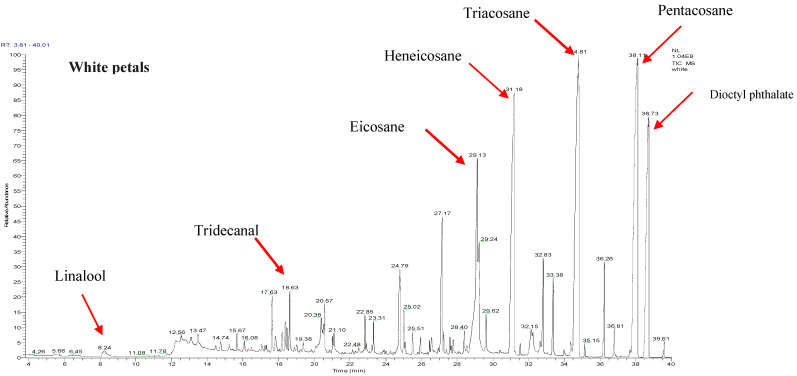

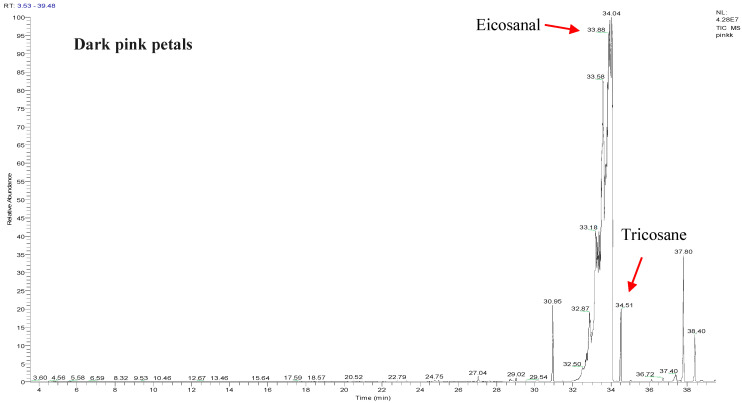
Total Ion Chromatogram (TIC) of white and dark pink *R. canina* L. petals.

**Figure 2:**
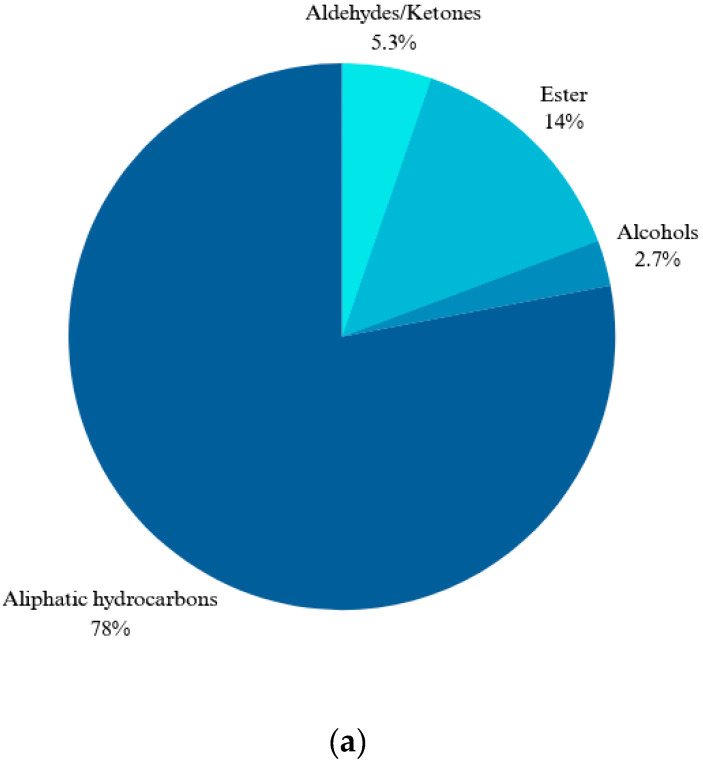

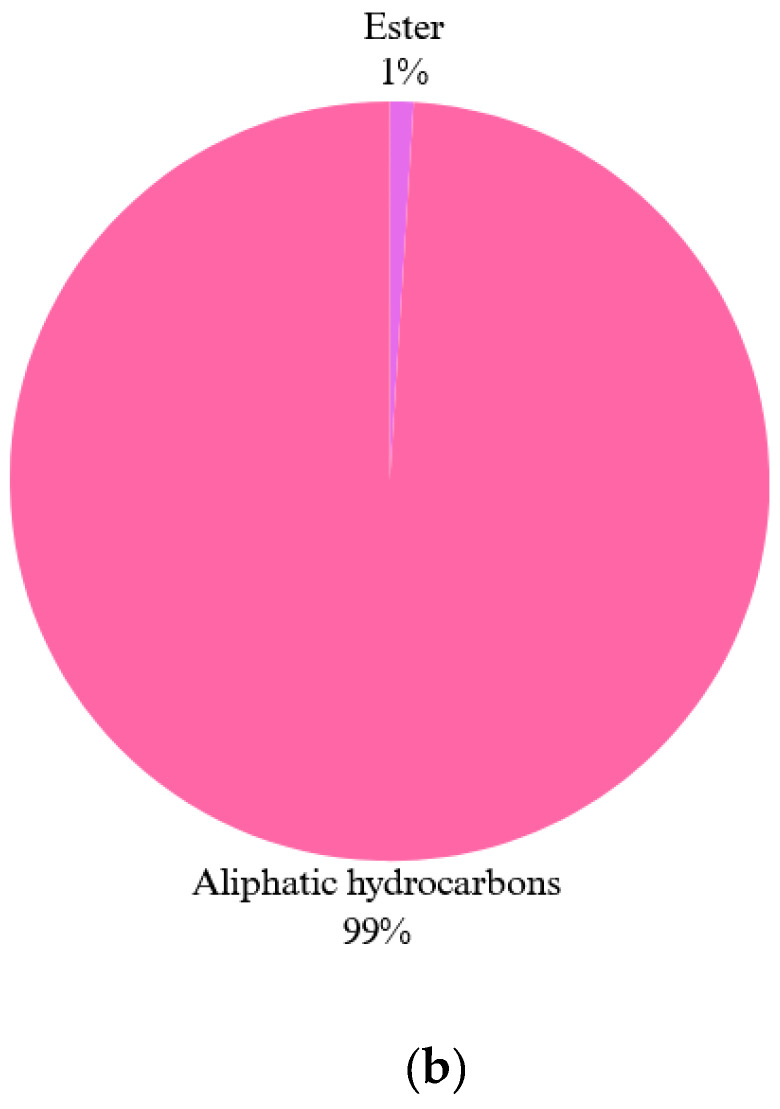
Percentage of chemical compositions of essential oils in white (**a**) and dark pink (**b**) petals.

The chemical composition of essential oil extracted from *R. canina* petals by hydrodistillation was analyzed by GC-MS. Two petal color variants, white and dark pink, were compared. The GC-MS analysis detected 32 and 15 compounds in the white and dark pink samples, respectively (Table 1 and Table 2). These compounds belonged to four classes: aliphatic hydrocarbons, aldehydes/ketones, alcohols, and esters. Aliphatic hydrocarbons predominated in both samples, accounting for 76.8% and 99% of the total oil composition in the white and dark pink samples, respectively. The white sample also contained two monoterpenes, linalool and geranial, and their corresponding acetate esters, citronellyl acetate and linalool acetate. Pentacosane was the most abundant hydrocarbon in the white sample (22.34%). Moreover, bis(2-ethylhexyl) terephthalate (DEHT), a natural diester of terephthalic acid and 2-ethylhexanol, was identified in the white sample. DEHT, also known as octyl, has a similar chemical formula and structure to the synthetic plasticizer bis(2-ethylhexyl) phthalate (DEHP) [32]. DEHT is a natural constituent of rose oil, as reported in previous studies [33,34,35,36,37]. Figure 1 indicates the Total Ion Chromatogram (TIC) of white and dark pink *R. canina* L. petals and Figure 2 shows the relative percentages of the volatile compounds in the two samples.

### 2.2. Total Phenolic and Flavonoid Content of the Petal Extracts

**Figure 3:**
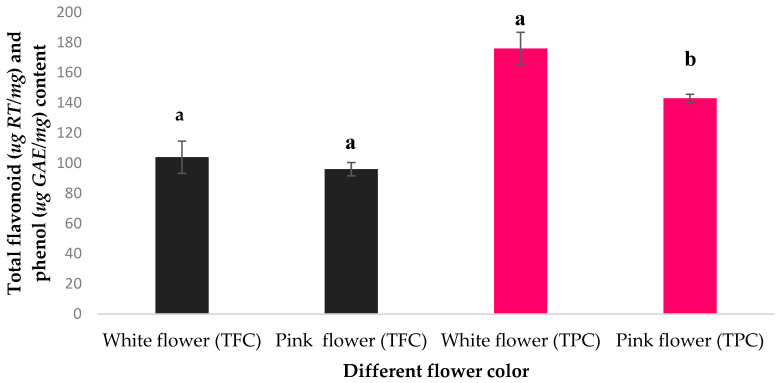
The amount of total flavonoid content (TFC) and total phenol content (TPC) in white and pink flowers, compared by Duncan’s multiple range test. Different letters (a and b) indicate significant differences at *p* < 0.05.

The Folin–Ciocalteu and aluminum chloride methods were used to measure the total phenolic content (TPC) and total flavonoid content (TFC) of the hydro-methanolic extracts of *R. canina* petals, respectively. The TPC and TFC values were expressed as micrograms of gallic acid equivalents (GAE) and micrograms of Rutin equivalents (RE) per milligram of dry weight (DW) of petals, respectively. The standard curve equations for TPC and TFC were (y = −0.0153 + 0.0066x) and (y = 0.0142 + 0.0008x), respectively. The TPC and TFC of the white-petaled and dark pink-petaled cultivars of *R. canina* were compared in Figure 3. The white-petaled cultivar had a significantly higher TPC than the dark pink-petaled cultivar (*p* < 0.05), while the TFC was slightly higher but not significantly different *p* > 0.05).

### 2.3. Total Anthocyanin Content (TAC)

The TAC of white and dark pink petals was determined using a spectrophotometric method. The results showed that dark pink petals had a significantly higher TAC than white petals. The mean TAC values were 9.12 µmol/g for dark pink petals and 3.21 µmol/g for white petals. The difference in TAC between the two types of petals could be attributed to the presence of different anthocyanin pigments, which are responsible for the color variation. Anthocyanins are water-soluble plant pigments that belong to the phenylpropanoid pathway. They have various biological functions and health benefits, which will be discussed in the next chapter.

### 2.4. Liquid Chromatography-Electrospray Ionization-Tandem Mass Spectrometry (LC-ESI-MS) Phenolic Profile of R. canina Methanolic Extract

**Figure 4:**
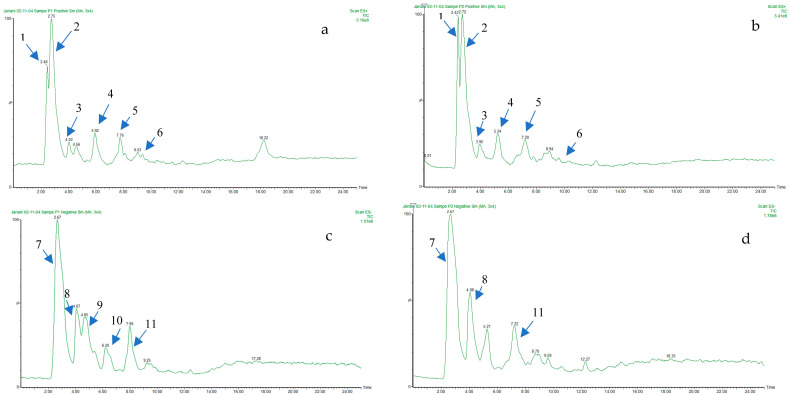
Total Ion Chromatograms of *R. canina* petals in positive and negative modes. The figure shows the chromatograms of white (**a**,**c**) and dark pink (**b**,**d**) petals in positive (**a**,**b**) and negative (**c**,**d**) modes, respectively. The peaks correspond to the compounds detected in the petals. The compounds are as follows: 1: Quercetin-3-O-rutinoside-7-O-glucoside, 2: Dihydroxy-dimethoxy flavone, 3: Gallic acid, 4: Cyanidin, 5: Quercetin-3-O-hexoside, 6: Kaempferol-3-O-rutinoside, 7: Kaempferol 3-O-(6″-malonyl-glucoside), 8: Tetra-O-galloyl-hexoside, 9: Myricetin, 10: Ellagic acid, and 11: Kaempferol-3-O-glucoside.

Phenolic compounds are a diverse group of plant metabolites that exhibit antioxidant activity and complex structure. Flavonoids are the most abundant phenolic compounds in plants and have various biological effects. *R. canina* is a plant species that contains high levels of flavonoids and other phenolic compounds in its petals. To identify and characterize the phenolic profile of *R. canina* methanolic extract, liquid chromatography-electrospray ionization-tandem mass spectrometry (LC-ESI-MS) was employed. This technique allows the separation and identification of phenolic compounds based on their retention time and mass fragmentation pattern in positive and negative ionization modes. The results were verified by comparing them with previous studies. The LC-ESI-MS analysis revealed that flavonoids, especially rutin, quercetin, kaempferol, and their derivatives, were the predominant phenolic compounds in *R. canina* methanolic extract. Gallic acid and ellagic acid were the main phenolic acids detected. Moreover, cyanidin, delphinidin, and pelargonidin were the major anthocyanins in white and dark pink petals, respectively. Figure 4 shows the total ion chromatograms of *R. canina* petals in both ionization modes. Table 3 summarizes the different phenolic compounds and their characteristics that were identified by LC-ESI-MS.

### 2.5. Gene Expression Analysis

**Figure 5:**
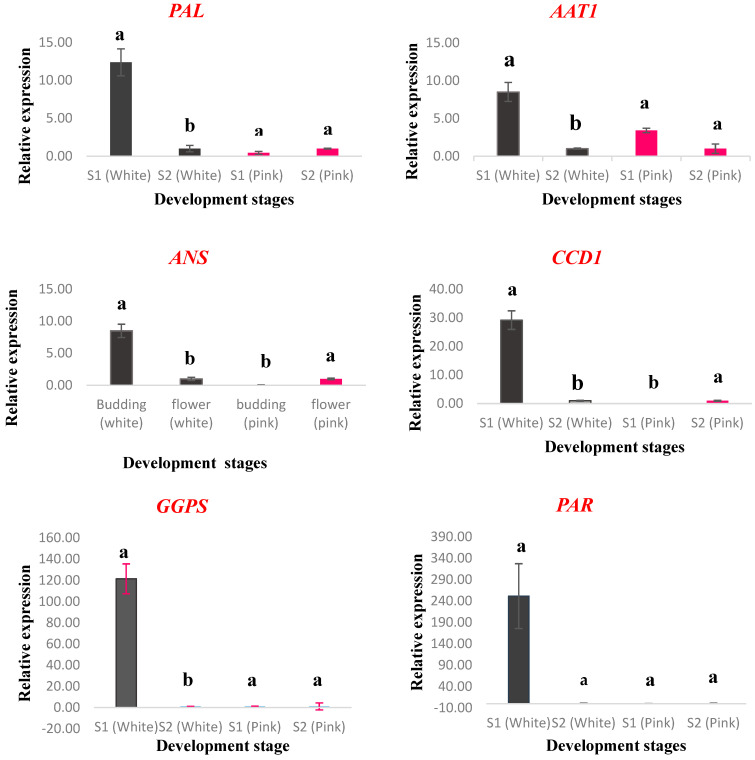

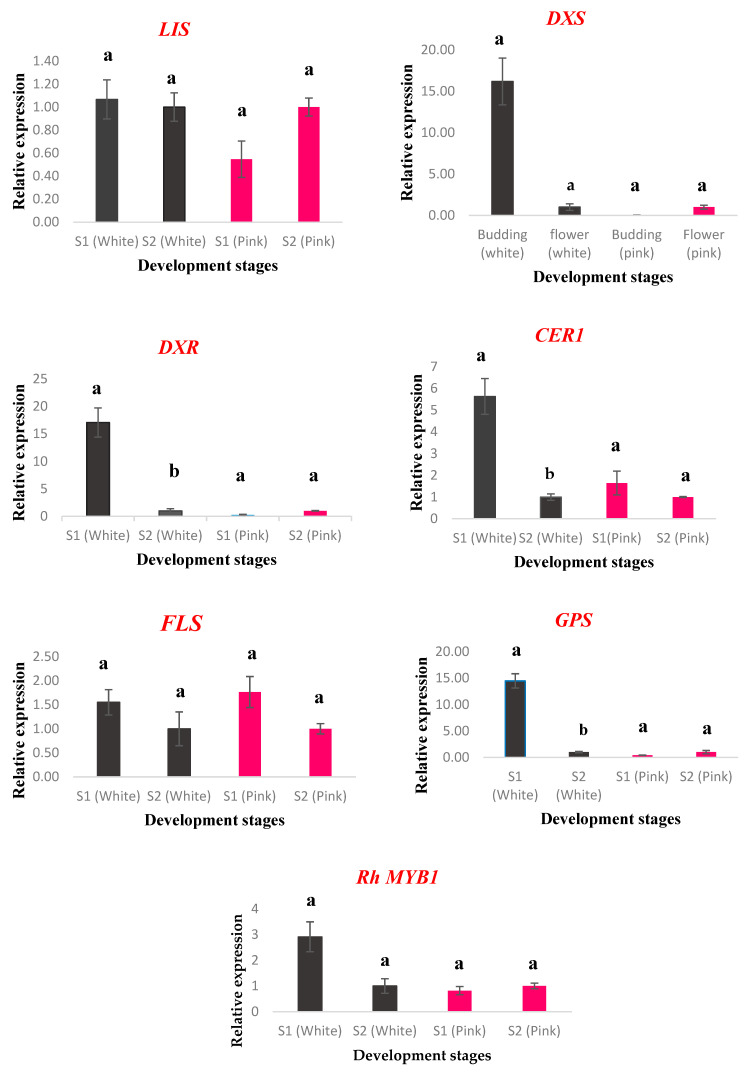
Relative expression of main genes which are involved in flower color and scent biosynthesis in rose flower. Different flower developmental stages including budding stage (S1) and open-flower stage (S2) was selected for this assay. The fold change expression was calculated after normalization to the beta-actin gene. The open-flower stage (S2) was selected as a reference for white and dark pink petals. Standard errors are indicated by vertical bars. The values of error bars are revealed mean ± SE, *n* = 3. Different letters (a and b) indicate significant differences at *p* < 0.05 between S1 and S2 for each petal color, as determined by one-way ANOVA and Duncan’s test.

The qRT-PCR method measured and compared the relative expression levels of 13 genes involved in floral color and scent biosynthesis at two key stages of flower development when scent emission occurs: budding and open-flower. The genes were *PAR*, *GPS*, *GGPPS*, *PAL*, *DXR*, *DXS*, *LIS*, *CCD1*, *AAT1*, *ANS*, *FLS*, *CER1*, and *RhMYB1.* Figure 5 shows the expression patterns of these genes at different stages.

White flowers had higher expression levels of all main genes at the budding stage than at the open-flower stage. The expression profiles of these genes gradually decreased as the flowers opened. Seven genes (*GPS*, *GGPPS*, *DXR*, *DXS*, *LIS*, *CCD1*, and *AAT1*) related to floral terpene volatile compound biosynthesis showed similar expression trends. Among them, *GGPS* had the highest expression level in white floral buds (121.38-fold), followed by *CCD1* (29.18-fold), *DXR* (17.06-fold), *DXS* (16.19-fold), and *GPS* (14.49-fold). Except for *FLS*, *DXS*, *LIS*, and *PAR*, the other genes in white flowers had significant differences in expression levels among different stages.

Dark pink petals had higher expression levels of the *ANS* gene, which participates in anthocyanin synthesis, than white petals. The expression of *ANS* and *CCD1* differed significantly in dark pink petals, while the other genes did not. The *ANS* gene had remarkably higher expression levels at white flower buds (8.50-fold) than at dark pink flower buds, followed by the *FLS* gene (1.55-fold). The *FLS* gene, which is the key gene for flavonol synthesis, had higher expression levels at the budding stage (1.77-fold) than at the open-flower stage in dark pink flowers. The *ANS* gene had the opposite trend: it had higher expression levels at the open-flower stage than at the budding stage in dark pink flowers. The *FLS* gene catalyzes flavanol biosynthesis, which peaks in the early stages of flower development before anthocyanin accumulation. The *ANS* gene catalyzes anthocyanin formation, such as pelargonidin and cyanidin.

In white petals, the expression of the *ANS* and *PAL* genes, which are related to anthocyanin biosynthesis, was the highest at the budding stage. In dark pink petals, the expression levels of *PAL* and *PAR* genes, which are also related to anthocyanin biosynthesis, were lower at the budding stage than at the open-flower stage. The *PAR* gene had the highest expression level (251.31-folds) at the budding stage of white flowers, followed by the *PAL* gene (12.38-folds). The *CER1* gene, which is involved in long-chain alkane synthesis in epidermal parts of flower petals, had higher expression levels in white flower buds (5.63-folds) than in dark pink flower buds. The *RhMYB1* gene, a key transcription factor that regulates the anthocyanin biosynthesis pathway, had consistent expression results with the main genes, especially the *ANS* gene. For white flowers, the *RhMYB1* gene had higher expression levels at the budding stage (2.91-folds) than at the open-flower stage. For dark pink flowers, the *RhMYB1* gene had lower expression levels at the budding stage than at the open-flower stage.

### 2.6. Cluster Analysis

**Figure 6:**
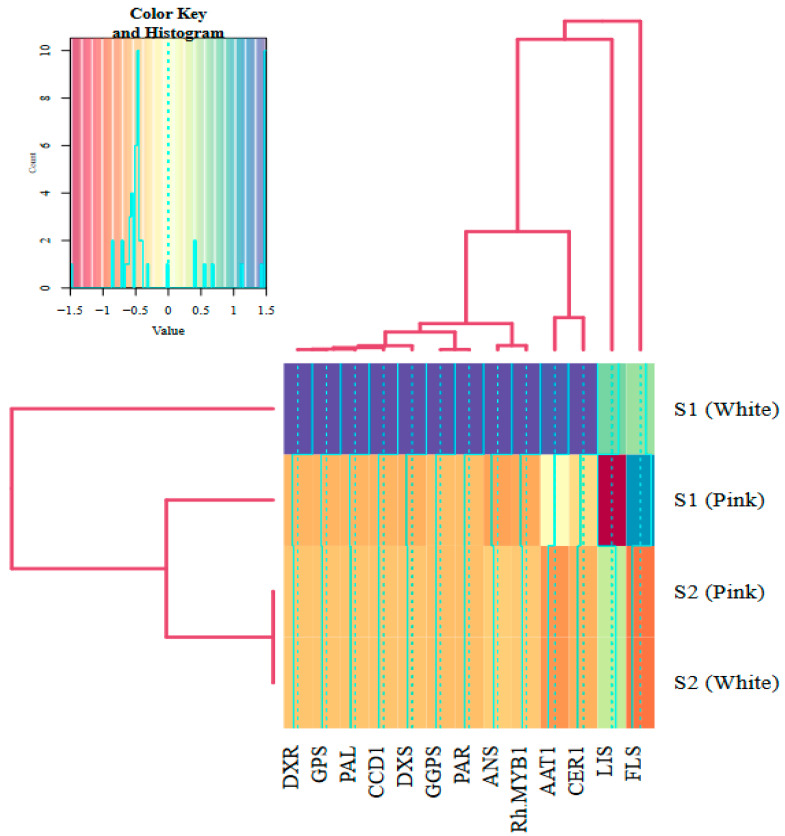
Heatmap and cluster analysis of gene expression in flower development stages.

As depicted in Figure 6, the correlation and cluster analysis revealed distinct groupings among the genes. The *ANS* and *RhMYB1* genes, along with *PAR*, were first grouped into a single cluster. In contrast, *GGPS*, *PAR*, and *DXS* formed another separate group. Furthermore, genes associated with the terpene biosynthesis pathway, such as *DXR*, *GPS*, *PAL*, *CCD1*, *DXS*, and *GGPS*, exhibited high similarity and were categorized within a similar cluster. Subsequently, these groups were amalgamated into the same cluster in the following step of the analysis. This clustering suggests a potential regulatory relationship and functional proximity within the color and scent biosynthesis pathway.

This figure presents a heatmap alongside cluster analysis, illustrating gene expression patterns related to flower color and scent across two distinct developmental stages: budding stage (S1) and open-flower stage (S2). The heatmap visualizes fold changes in gene expression, with darker shades indicating higher expression levels and lighter shades denoting lower levels. Hierarchical clustering is depicted through dendrograms positioned above and to the left of the heatmap, representing the fold changes and various treatments, respectively. The clustering employs the Ward method, highlighting similarities in gene expression profiles.

## 3. Discussion

*R. canina* is a rare type C wild rose that displays distinct features, such as high monoterpene levels at the bud stage, low geraniol content, short floral longevity, and white petals [38]. Monoterpenes are volatile metabolites that perform various roles in plant physiology, such as mitigating oxidative damage by reactive oxygen species (ROS), modulating isoprenoid hormone levels, and extending floral lifespan [39]. The decrease in monoterpene levels at the senescence stage may account for the reduced floral longevity of type C roses, as hypothesized by [38]. This is because monoterpenes may have antioxidant properties and protect the petals from ROS-induced oxidative stress during senescence [40]. Furthermore, monoterpenes may influence the levels of abscisic acid (ABA), a plant hormone that controls senescence and wilting [41]. Hence, lower monoterpene levels may lead to higher ROS and ABA levels, which may accelerate the senescence process and shorten the floral lifespan. These characteristics were corroborated by the findings of this study, which unveiled new aspects of the volatile profile of *R. canina* essential oil. The examination of the essential oil revealed that linalool was the dominant monoterpene compound in dog rose oil, followed by geranial. Linalool is an acyclic monoterpene alcohol with a fresh and sweet odor that belongs to the fruity odor group [42].

Geranial is a monoterpene aldehyde that adds to the floral scent of roses [43]. The relatively high proportion of these compounds in *R. canina* essential oil suggests that this species may have an appealing and fruity fragrance that could lure pollinators and consumers. However, the findings of this study were in line with a previous study that reported a decline in monoterpene synthesis during flower aging [44]. The diminution of monoterpenes at the senescence stage may be linked to the shorter floral longevity of type C roses, as proposed by Dani et al. [38]. Moreover, the findings showed that *R. canina* essential oil contained a substantial amount of long-chain hydrocarbons, particularly nonadecane and heneicosane, which caused the oil to solidify at room temperature. This observation concurs with [45], who reported that *R. canina* oil had a high percentage of long-chain hydrocarbons and a low percentage of geraniol compared to other rose species.

The stability and quality of *R. canina* essential oil may depend on the presence of long-chain hydrocarbons, which have different properties from short-chain hydrocarbons. Long-chain hydrocarbons exhibit lower volatility and higher viscosity, which may influence the evaporation rate and oxidation susceptibility of *R. canina* essential oil [46,47]. These factors may alter its chemical composition and aroma over time. Moreover, long-chain hydrocarbons may affect the biological activity and safety of *R. canina* essential oil, as they may have different interactions with biological molecules than short-chain hydrocarbons [48]. Thus, the implications of long-chain hydrocarbons for the stability and quality of *R. canina* essential oil, as well as their potential applications in various domains, should be investigated. This study demonstrated that the chemical composition of *R. canina* flowers is affected by the number and color of petals. Aromatic hydrocarbons were more abundant in flowers with four single petals than in those with more petals, which contained higher levels of terpenoids. This is in accordance with previous researches by [1,49], who reported that the number of petals modulates the biosynthesis and emission of scent compounds in roses. This study also revealed that white petals contained more aromatic compounds than dark pink ones, indicating that petal color influences the floral scent profile as well. This is congruent with the findings of [38,50,51,52,53], who observed that light-colored flowers produce more intense and diverse scents than dark pink flowers. Future studies could examine the biological activities and pharmacological properties of *R. canina* essential oil, as well as its possible applications in perfumery, cosmetics, or aromatherapy. The results also showed that the white petals had higher levels of both phenolic and flavonoid compounds than the dark pink petals, suggesting a possible association between petal color and phytochemical content. These results are consistent with a previous study that found higher phenolic and flavonoid contents in white *R. canina* flowers compared to other colors [54]. Phenolic and flavonoid compounds are known to possess various biological activities, such as antioxidant, anti-inflammatory, anti-microbial, and anti-cancer properties [55]. The white petals of *R. canina* have higher flavonoid content than the dark pink petals, which may indicate their higher potential for medicinal and cosmetic applications. Flavonoids are known to have antioxidant, antimutagenic, and anticarcinogenic effects [56,57].

The white petals of *R. canina* var. assiensis had a higher total flavonoid content (163.3 mg/100 g frozen pulp) than other rose species (101.3–143.7 mg/100 g frozen pulp) [58]. The flavonoid content of *R. canina* fruits was much lower (41 mg/100 g dry matter) than that of white petals [59]. Thus, the petal color of flowering plants may reflect their phytochemical composition and bioactivity, besides their ornamental value. Different pigments, such as anthocyanins, flavonoids, and carotenoids, confer various colors to petals and also exhibit biological activities, such as antioxidant, anti-inflammatory, and enzyme inhibition effects. Hence, choosing the best cultivars with optimal petal colors could offer multiple advantages for different applications, such as herbal remedies, natural dyes, or landscaping. Previous studies have demonstrated the relationship between petal color and phytochemical content in different species of flowering plants. For instance, *Paeonia delavayi* showed significant differences in anthocyanin and flavonoid content and antioxidant and enzyme inhibition activities among purple, red, and yellow petals [60]. Similarly, marigold flowers with different petal colors from off-white to deep orange had varying amounts of carotenoids, phenolics, flavonoids, and antioxidant potential [61]. These findings indicate that petal color could be a useful criterion for selecting the most appropriate cultivars of flowering plants for different purposes. Moreover, these results may improve the understanding of the biosynthesis and regulation of phenolic and flavonoid compounds in plants, which are affected by various factors such as genetic, environmental, and developmental factors [62]. This study examined the relationship between petal color and total TAC in two cultivars of chrysanthemum with different petal colors: white and dark pink. The expression levels of the *ANS* gene, which is a key enzyme in anthocyanin biosynthesis, were also assessed in the petals at different developmental stages. The results revealed that dark pink petals had significantly higher TAC and *ANS* expression than white petals, especially at the blooming stage. This implies that anthocyanin synthesis and accumulation are increased in the dark pink petals through the phenylpropanoid pathway. These findings are in agreement with previous reports that showed a positive correlation between petal color intensity and TAC in various plant species [28]. However, it was also noticed that white petals had relatively high *ANS* expression at the budding stage, suggesting that anthocyanin biosynthesis is initiated but not completed in these petals. This could be due to the absence of other enzymes or cofactors required for anthocyanin production, or the presence of inhibitors or degrading factors that prevent anthocyanin accumulation. Further studies are needed to clarify the molecular mechanisms underlying the color difference between the white and dark pink petals.

Petal color in plants is influenced by anthocyanins, which are plant pigments that belong to the flavonoid family. Anthocyanins have various health benefits for humans and animals, such as protecting against oxidative stress, inflammation, cancer, diabetes, and neurodegeneration [63]. They also affect the attraction of pollinators, which influences plant reproduction and adaptation. The phenolic composition of *R. canina* petals, which are rich in flavonoids, especially Rutin, quercetin, kaempferol, and their derivatives, as well as phenolic acids, such as gallic acid and ellagic acid, was analyzed in this study. These phenolic compounds have been reported to have antioxidant, anti-microbial, anti-inflammatory, and anticancer activities. Therefore, *R. canina* petals could be a potential source of natural bioactive compounds for health and industry applications. The phenolic composition of *R. canina* petals was consistent with previous research [64]. These compounds could be unique to *R. canina* or specific to the geographical origin or cultivar of the plant. Further studies are needed to confirm their identity and biological activities. The phenolic content of white and pink petals, which differed in color due to the presence and concentration of anthocyanins, a subclass of flavonoids, was also compared in this study. Anthocyanins are responsible for the red, purple, and blue colors in plants, while flavonols, such as quercetin and kaempferol, are responsible for the yellow color [65]. White petals had less anthocyanins and more flavonols than pink petals, resulting in higher phenolic content. This finding was in agreement with previous studies that showed a negative correlation between anthocyanin content and total phenolic content in rose petals [66]. The expression levels of several genes related to the fragrance and color of roses were also investigated in this study. The results showed that the *GPS*, *CCD1*, *RrAAT1*, *LIS*, *DXS*, *DXR*, and *PAR* genes were significantly upregulated in fragrant roses compared to non-fragrant ones [67,68]. Moreover, MYB, bHLH, and WD40 transcription factors were differentially expressed between white and pink roses (Wei et al., 2007) [69]. These results were consistent with previous studies that have identified these genes and transcription factors as key players in the biosynthesis of volatile compounds and pigments in roses and other plants [3,24,50,69,70,71,72,73,74,75,76].

This study explores the molecular mechanisms that cause variation in rose fragrance and color, which are important for breeding and consumer preferences. A putative gene, *RhMYB1*, was found to be expressed in wild rose species with pleasant fragrance compounds, but not in non-aromatic roses [75]. The phenolic content and composition of rose petals depend on their color and genotype. Previous studies have shown that darker-colored petals, such as red and purple, have more anthocyanins than lighter-colored ones, such as white and yellow [77]. However, lighter-colored petals have more flavonoids than darker-colored ones, resulting in higher total phenolic content. This is consistent with other studies that reported a negative correlation between anthocyanin and total phenolic content in rose petals [78]. The effects of different light conditions on the phenolic content and composition of rose petals from different genotypes were investigated in this study. Rose petals are also known for their fragrance, which is mainly derived from volatile compounds such as monoterpenes, sesquiterpenes, and carotenoids [67]. Several genes involved in the biosynthesis of these compounds have been identified and isolated from roses, such as GGPPS, *GPS*, *RrAAT1*, *DXS*, *DXR*, *LIS*, *CCD1*, *PAR*, and *PAL* [68,73,79,80]. These genes encode enzymes that catalyze different steps of the metabolic pathways leading to various volatile compounds. For example, *GPS* synthesizes *GPP*, a precursor of C10 monoterpenoids; *CCD1* cleaves carotenoids to produce apocarotenoids; *RrAAT1* converts terpen alcohols to acetic esters; and *LIS* synthesizes linalool from *GPP* [3,67,73,81]. The expression levels of these genes in rose petals under different light conditions were measured and correlated with the volatile profiles of the petals. The color and fragrance of rose petals are also regulated by transcription factors (TFs) that modulate the expression of structural genes involved in the biosynthesis of pigments and volatiles. Some of the TFs that play a key role in this process are MYB, bHLH, and WD40 [74]. These TFs form complexes that bind to specific DNA sequences and activate or repress the transcription of target genes [72]. For instance, MYB-TFs regulate anthocyanin and flavonoid biosynthesis by activating structural genes such as *CHS*, *F3H*, *DFR*, *ANS*, and *UFGT* [82]. Similarly, bHLH and WD40 TFs interact with MYB-TFs to regulate anthocyanin biosynthesis by forming MBW complexes [77,78]. Moreover, *RhMYB1* is a putative TF that is expressed in aromatic roses but not in non-aromatic roses, suggesting its role in regulating volatile biosynthesis [75]. The expression levels of these TFs in rose petals under different light conditions were analyzed and examined their relationship with the pigment and volatile contents of the petals.

The developmental stages of flowers significantly affect the volatile compounds they emit, as previous studies have shown [67,83,84]. The fragrant compounds also vary considerably throughout the flower development. The authors of [85] examined the volatile components of *R. odorata* and *R. chinensis* and reported that the flowers with four single petals had higher amounts of total volatile compounds in the first stage of development and then decreased sharply. These results agreed with the findings of the phytochemical investigation. The authors of [86,87] found a positive correlation between the expression of *DXR*, *DXS*, *LIS*, and *AAT1* genes and the rate of monoterpene biosynthesis pathway in the rose flower. They inferred that higher *DXR* expression could be associated with higher monoterpene accumulation, implying that the *DXR* gene has a critical role in monoterpene biosynthesis in *R. canina* petals. Other studies explored the link between monoterpene content in Artemisia annua and the expression of key genes in the MEP and artemisinin biosynthesis pathway, which is the main monoterpene biosynthesis pathway in plants. They used qRT-PCR to verify that the upregulation of the main MEP pathway genes led to more monoterpenes in plant tissues. Ahmadian [88] observed changes in the relative gene expression and metabolites of terpenoids and phenylpropanoids compounds during different flower developmental stages in tuberose. This research emphasized the importance of timing the developmental stages when studying the regulation of secondary metabolites. Ranjbar [89] analyzed the expression patterns of artemisinin biosynthesis genes in eight different Artemisia species at three developmental stages. The study detected significant differences in the expression levels of these genes among species and developmental stages. The expression of *FLS* gene, which catalyzes flavanol synthesis, was higher at the budding stage than at the open-flower stage in both cultivars, while the expression of *ANS* gene, which catalyzes anthocyanin formation, was higher at the open-flower stage than at the budding stage in the dark pink cultivar, but not in the white cultivar. The differential expression of anthocyanin biosynthetic genes in the petals of *R. canina* explains the molecular basis of the color variation between the dark pink and white cultivars. Among these genes, *ANS* was the most significantly upregulated gene in the dark pink petals, indicating its key role in regulating the anthocyanin accumulation in this tissue. This finding offers a valuable target for breeding strategies aimed at improving the anthocyanin production and quality in *R. canina* and other ornamental plants. Flavanol synthesis is more active in the early stages of flower development, before anthocyanin accumulation, which is consistent with previous studies. The higher expression of *ANS* gene in the dark pink cultivar at the open-flower stage may be related to the increase in vacuolar pH during flower opening, which enhances anthocyanin stability and color intensity [90,91]. White petals of *R. canina* gradually fade to pure white during flower development due to downregulation of anthocyanin biosynthesis-related genes [92].

Ahmadian [88] reported that the relative gene expression and metabolites of terpenoids and phenylpropanoids compounds varied during different flower developmental stages in tuberose. This finding underscores the need to account for the timing of developmental stages in the analysis of secondary metabolites. [89] examined the expression patterns of genes involved in artemisinin biosynthesis in eight Artemisia species at three developmental stages. They detected significant differences in the expression levels of these genes among species and developmental stages. This result corroborates the study of (Ahmadian et al., 2018) [88], which showed that *PAR* gene accumulation was higher in flowers at the budding stage due to the presence of fresh stamen, while it was lower in open flowers. This result was consistent with the GC-MS data analysis, which indicated that the essential oil with the highest percentage of aliphatic hydrocarbons was obtained from flowers at the budding stage. This study also revealed that phenylalanine metabolism differed between white and pink roses, affecting their fragrance production. White flowers had higher levels of 2-phenylethanol, which is derived from phenylalanine at the initial step of the phenylpropanoid biosynthesis pathway. Pink roses, however, used phenylalanine to produce anthocyanins, which gave them their color. Consequently, the expression of genes related to phenylpropanoid biosynthesis, such as *PAL* and *PAR*, was lower in pink roses than in white flowers. Nevertheless, *PAL* also contributed to anthocyanin biosynthesis, which might influence the trade-off between fragrance and color production in different rose varieties. Comparative transcriptomic analysis indicated that *GGPPS* and *PAR* were the key regulatory genes for monoterpene and phenylpropanoid biosynthesis, respectively, in white flowers at the budding stage.

## 4. Materials and Methods

### 4.1. Plant Materials

The study used two color morphs of *R. canina* flowers, white and dark pink, which are endemic to the mountainous areas of Taleghan, Alborz province, Iran. The flowers were collected from a wild population at 36°10′18.8″ N and 50°46′12.7″ E and identified by their morphological features of leaves, fruits, and flowers, based on the methods of [93,94]. A voucher specimen (number 006962) was deposited in the Herbarium Instituti Agronomici KeredJensis (HIAK) by a botanist from the horticulture department of the University of Tehran (Dr. Vahideh Nazeri Jounghani). The flowers were harvested in the evening (6:00 p.m.) before sunset, which previous studies [95,96] indicated is the optimal time for volatile oil content.

**Figure 7:**
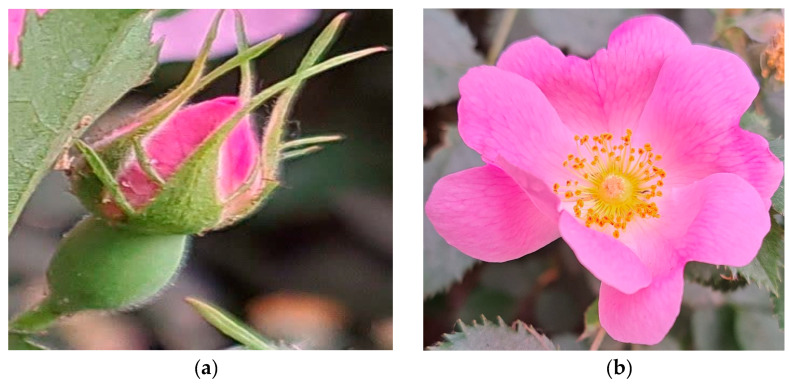

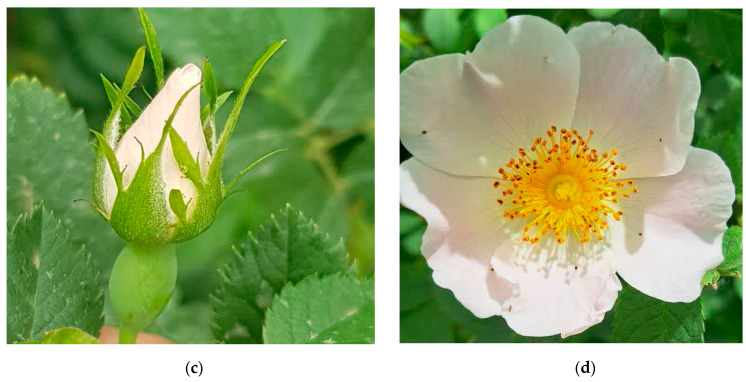
Different developmental stages of dark pink and white *R. canina* flowers. Budding stage (S1) (**a**,**c**); open-flower stage (S2) (**b**,**d**).

White and dark pink shrub samples were obtained from the same plant. For each sample, three technical replicates were prepared for molecular analysis. The flowers were picked at two stages of floral development that were important for scent emission: the budding stage (S1) and the open-flower stage (S2) [97]. The development stages were determined by measuring the flower length, weight, and diameter, and by observing the sepal shape and the pollen color and texture. In the budding stage (S1), the sepals were closed but the petal colors were visible. In the open-flower stage (S2), the pollen was light yellow and the petals were fresh and dark. These two stages had higher scent emission than the senescence stage, based on phenotypic characteristics (Figure 7). To prevent RNA degradation, the fresh samples were immediately frozen in liquid nitrogen and stored at −80 °C until further analysis. For dried petals powder preparation, the petals were detached and dried in a cool and dark place, then ground into powder [98].

### 4.2. Extraction of the Essential Oils

To extract *Rosa* essential oils (EOs), the fresh petals were carefully separated from the other parts of the flower [99]. Around 200 g of fresh petals from both white and dark pink flowers were weighed and used for the extraction process. The EOs extraction was carried out using the hydrodistillation method, which involves heating water and plant material in a closed system and condensing the vapor containing the volatile compounds [100]. This method was chosen because it is simple, inexpensive, and widely used for extracting EOs from various plants [101]. The extraction was performed using a 2 L Clevenger apparatus for a duration of 4 h. The extracted EOs were stored in a dark glass vial and kept at a low temperature of −4 °C to prevent evaporation and loss of EOs until GC-MS analysis.

### 4.3. Chemical Compositions of the R. canina Essential Oil by GC-MS Analysis

The chemical composition of the essential oils (EOs) was analyzed by GC-MS using a ThermoQuest Finnigan TRACE MS system with a quadrupole mass analyzer, as described by Naquvi et al. [102]. The GC column was a non-polar DB-5 fused silica capillary (30 m × 0.25 mm × 0.25 µm film thickness), and the carrier gas was helium at a flow rate of 1.1 mL/min. The injection volume was 1 µL, and the split ratio was 1:10. The injector and detector temperatures were 250 °C and 280 °C, respectively. The oven temperature was programmed from 60 °C to 250 °C at 5 °C/min, and then held at 250 °C for 10 min. The mass spectra were recorded in the electron ionization mode at 70 eV, scanning from 40 to 460 *m*/*z*, with a scan time of 0.4 s. The peak areas of the GC-MS chromatograms were integrated using the Xcalibur software (version 2.0, Thermo Fisher Scientific, Waltham, MA, USA). The peak identification was based on the comparison of the retention indices (RI) and the mass spectra of the compounds with those of authentic standards and literature data. The RI values were calculated by interpolation using a homologous series of n-alkanes (C8–C20) as reference compounds, according to the following formula:

RI: 100 × Cn + 100 × (T_n_ − S_n_)/ (S_n+1_ − S_n_)

where RI is the retention index of the compound, Cn is the number of carbon atoms in the n-alkane eluting before the compound, Tn is the retention time of the compound, S_n_ is the retention time of the n-alkane eluting before the compound, and S_n+1_ is the retention time of the n-alkane eluting after the compound. The relative percentages of the identified compounds were calculated based on the peak areas without using correction factors. The GC-MS data were also analyzed using the Kyoto Encyclopedia of Genes and Genomes (KEGG) [103] and MetaCyc [104] databases to identify the key genes involved in the biosynthesis of floral aromatic compounds. The metabolic pathways and enzymes were retrieved from the databases using the compound names and structures as queries [105,106].

### 4.4. Measurement of Total Phenol Content

The total phenolic content of white and dark pink *R. canina* petals was determined by the Folin–Ciocalteu method, following the procedures of [107,108]. The samples were analyzed in triplicate and their absorbance was measured at 760 nm using a microplate reader (Epoch, BioTek, Winooski, VT, USA) with Gene 5 software. The results were expressed as mg of gallic acid equivalents per g of dry weight. A completely randomized design was used to analyze the data with SAS 9.4 software (SAS Institute Inc., Tokyo, Japan). Duncan’s test was performed to compare the means at the significance levels of 5% (*p* ≤ 0.05) and 1% (*p* ≤ 0.01).

### 4.5. Measurement of Total Flavonoid Content

The spectrophotometric assay of Popova et al. [109] was applied to determine the total flavonoid content. This method relies on the complexation of aluminum ion with the carbonyl and hydroxyl groups of the flavonoid. The flavonoid content was expressed as rutin equivalents, using rutin as a standard. An Elisa system microplate reader (Epoch, BioTek) equipped with Gene 5 software, which is a device that measures the absorbance of a solution, was used to perform the assay at 510 nm [110]. The experiment was replicated three times for accuracy. SAS 9.4 software, which is a statistical software that can conduct various tests and analyses, was used to analyze the data with a completely randomized design (CRD). The mean values were compared by Duncan’s test at 5% and 1% significance levels (*p* ≤ 0.05 and *p* ≤ 0.01, respectively).

### 4.6. Measurement of Total Anthocyanin Content

The method of [111] was used to quantify the anthocyanin content of white and dark pink petals. Acidified methanol (1%, *v*/*v*; methanol/HCl = 99:1) was used to extract fresh petals (0.1 g) in 10 mL of solvent. The extract was centrifuged at 4 °C and 4000× *g* for 15 min and the supernatant was stored overnight at 5 °C in the dark. The supernatant was then filtered through a membrane filter and its absorbance at 530 nm was measured using a UV–VIS spectroscopy (Shimadzu, Kyoto, Japan, model UV-1601) with distilled water as a blank.

### 4.7. UPLC–Electrospray Ionization Mass Spectrometry (ESI-MS) Analysis

The methanolic extract of *R. canina* was analyzed for polyphenolic compounds, including phenolic acid and flavonoid components, by LC-*ESI-MS* [112,113]. The LC system was a Waters Alliance 2695 equipped with a C18 column (4.6 mm × 150 mm, 5 μm) maintained at 35 °C. The mobile phase consisted of acetonitrile with 0.1% formic acid (solvent C) and water with 0.1% formic acid (solvent B). The flow rate was 0.30 mL min^−1^. The MS system was a Micromass Quattro micro API operating in ESI mode with the following parameters: cone voltage, 20 V; capillary voltage, 4 kV; extractor, 2 V; RF lens, 0.2 V; collision energy, −eV; gas nebulizer, N_2 (grade 5); flow gas, 200 L/h; source temperature, 120 °C; desolvation temperature, 350 °C. The sample was filtered through a MILLEX GV membrane filter (0.22 μm pore size, Millipore, Burlington, MA, USA) before injection.

### 4.8. RNA Isolation and Quantitative Real-Time PCR (qRT-PCR)

**Figure 8:**
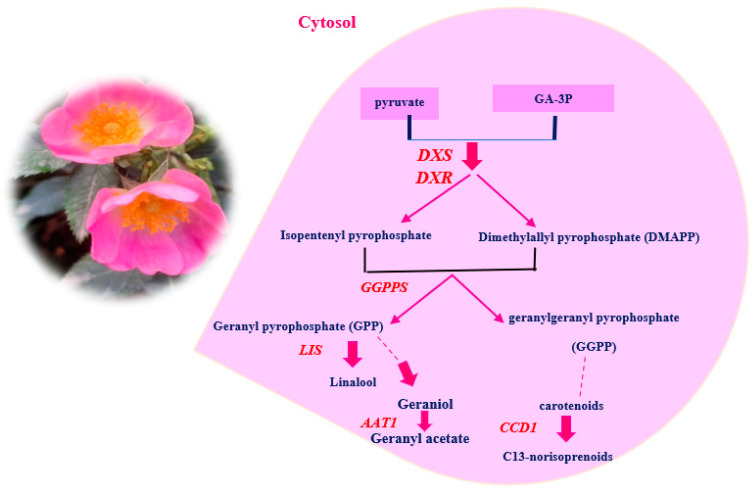
Schematic representation of the MEP pathway for terpenoids biosynthesis in roses. G3P, glyceraldehyde-3-phosphate; *DXS*, 1-deoxy-d-xylulose-5-phosphate synthase; *DXR*, 1-Deoxy-D-xylulose 5-phosphate reductoisomerase; *LIS*, linalool synthase; *AAT1*, aromatic amino acid aminotransferase; *CCD1*, carotenoid cleavage dioxygenase 1.

**Figure 9:**
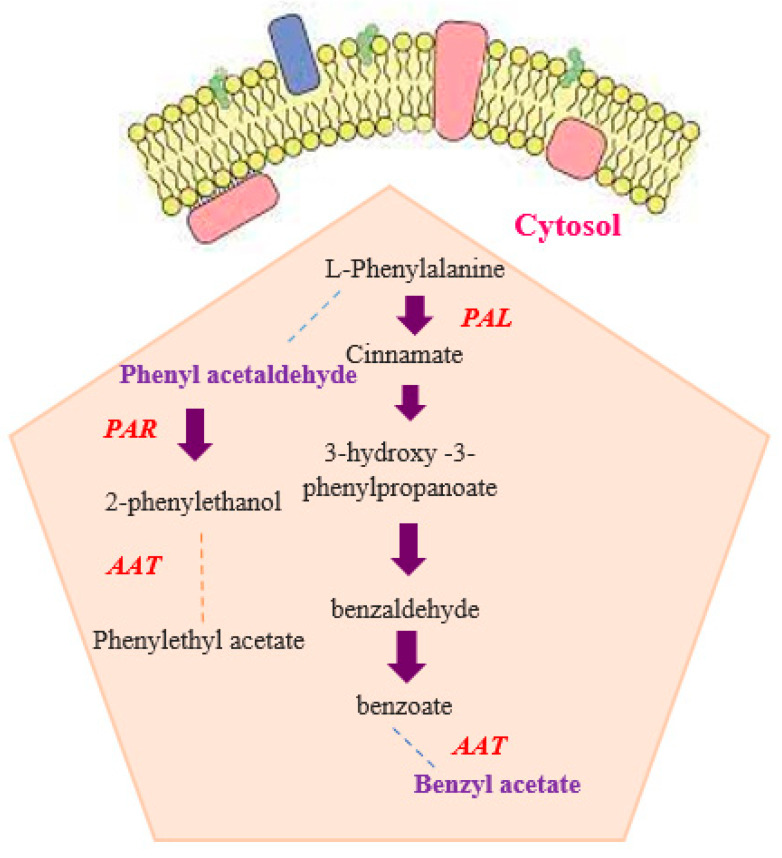
Schematic illustration of phenylpropanoids/benzenoids biosynthesis in rose flowers. *PAL*-phenylalanine ammonia lyase; *PAR*-phenyl acetaldehyde reductase; *AAT*-alcohol acetyltransferase.

For expression analysis of genes involved in flower color and scent, approximately 200 mg of petal sample was used. The samples were ground into a powder in liquid nitrogen, and total RNA was extracted using a modified CTAB method [114,115]. Genomic DNA contamination was eliminated using *DNase I*, RNase-Free DNase Set (Fermentas, Waltham, MA, USA). The quality and quantity of RNA samples were assessed using agarose gel electrophoresis and Nanodrop ND-1000, respectively [116,117]. To synthesize the first strand complementary DNA (cDNA), 1 μg of total RNA was converted into cDNA using the cDNA synthesis kit (A101161, Reverse Transcription Kit made by Parstous Iran) following the manufacturer’s instructions. The main genes identified by GC-MS data analysis and literature review were selected (Figure 8 and Figure 9).

The online version of primer3 software [118] was used to design the corresponding primers for *PAL*, *FLS*, *MYB1*, *PAR*, *GPS*, *GGPS*, *ANS*, *CCD1*, *LIS1*, *AAT1*, *DXR*, *DXS*, and *CER1* genes. The primer efficiency was checked using the Oli-go-analyzer tool and NCBI/Primer-BLAST software (https://www.ncbi.nlm.nih.gov/tools/primer-blast/). Table 4 lists the sequences of the forward and reverse primers used for qRT-PCR. The beta-actin gene served as a housekeeping gene for normalizing the expression of target genes. QIAGEN’s real-time PCR system was used to perform qRT-PCR following the manufacturer’s instructions [119,120]. Each reaction consisted of a 15 μL mixture containing 7.5 μL of SYBR green Master Mix 2X (ROX), 4.7 μL of RNase-free H_2_O, 2 μL of cDNA, 0.4 μL of forward primer, and 0.4 μL of reverse primer. The qRT-PCR thermal and temporal profiles were set to 15 min at 95 °C for initial denaturation, followed by 40 three-step cycles of amplification: 15 s of denaturation at 95 °C, 20 s of annealing at 56–59 °C (depending on the specific annealing temperature for each primer pair according to the gradient PCR results), and 20 s of extension at 72 °C. The 2^−ΔΔCT^ method [121] was used to calculate the relative expression levels of mRNAs. SAS 9.4 software was used to analyze the gene expression results based on a Completely Random Design (CRD). Duncan’s test was applied to examine the mean values of significance at the 5% (*p* ≤ 0.05) and 1% (*p* ≤ 0.01) levels.

To examine the patterns of similarity among the gene expression results, cluster analysis was performed using the R package. A heatmap was generated to visualize the clustering results based on the Ward distance method. The heatmap indicates the degree of association between the flower color and scent biosynthesis pathway by using a color scale [122,123].

## 5. Conclusions

This study examined the molecular and biochemical basis of petal color and scent variation in *R. canina*, a wild rose species with multiple medicinal and cosmetic uses. The results showed that *R. canina* petals produce a range of bioactive compounds that have positive effects on human health and well-being, such as flavonoids, anthocyanins, and volatile organic compounds. The white and dark pink petals differed significantly in their TPC, TFC, and TAC, with the white petals having higher TPC and TFC and the dark pink petals having higher TAC. The gene expression analysis revealed that most genes involved in petal color and scent biosynthesis were more highly expressed in the white buds than in the dark pink open flowers, except for anthocyanin synthase (*ANS*) and its regulator *RhMYB1*, which had similar expression levels in both flower colors. These findings indicate that *R. canina* flowers are rich sources of essential oils and that anthocyanin synthesis may be subject to feedback inhibition in white flowers. This study also demonstrated the genetic and metabolic diversity of *R. canina* petals and highlighted their potential as a valuable resource of natural products with high added value. The study also compared the results with those from other roses or plants and revealed that *R. canina* petals have distinctive characteristics that set them apart from other plant-derived compounds. For instance, the main phenolic compounds were flavonoids, especially quercetin, kaempferol, and their derivatives. Gallic acid and quinic acid were also detected as the predominant phenolic acids in *R. canina* petals. The white-petaled cultivar had a higher content of fragrant rose flower essential oil, which has been reported to possess anti-inflammatory, antioxidant, and antimicrobial properties. Thus, this study contributed to the understanding of the biology and biotechnology of *R. canina* petals and create new possibilities for their optimal exploitation and utilization. The study also suggested the need for further research on the biology and biotechnology of *R. canina* petals, such as the identification of the regulatory factors and the functional characterization of the genes, as well as the evaluation of the safety and efficacy of the bioactive compounds. This study lays the foundation for improving *R. canina* floral traits using metabolic and genetic engineering.

## Figures and Tables

**Table 1:** Percentage composition of the essential oils isolated from white *R. canina* petals.

No.	Compound Name	RI	Percentage Peak Area
1	Nonanal	1116	0.21
2	Linalool	1118	0.31
3	Linalool acetate	1268	1.06
4	Geranial	1279	0.82
5	Undecanal	1313	0.14
6	Citronellyl acetate	1352	0.24
7	Tetradecane	1397	0.21
8	Dauca-5,8-diene	1472	0.91
9	γ-Himachalene	1480	0.25
10	2-Tridecanone	1497	0.3
11	Isodaucene	1504	0.41
12	Tridecanal	1515	0.83
13	Dodecanoic acid	1588	1.03
14	Hexadecane	1596	0.73
15	Heptadecane	1696	0.48
16	Pentadecanal	1718	0.41
17	Benzyl benzoate	1789	2.03
18	Octadecane	1800	0.54
19	Nonadecane	1903	3.49
20	Eicosane	2003	8.16
21	Hexadecenoic acid	2009	2.77
22	Octadecanal	2029	0.48
23	Heneicosane	2114	13.84
24	Linoleic acid	2166	0.73
25	4 Docosane	2203	1.69
26	Eicosanal	2234	1.26
27	Triacosane	2317	19.74
28	Tetracosane	2396	1.61
29	1-Docosanol	2432	0.32
30	Pentacosane	2496	22.34
31	Dioctyl phthalate	2532	12.49
32	Hexacosane	2595	0.17

**Table 2:** Percentage composition of the essential oils isolated from pink *R. canina* petals.

No.	Compound Name	RI	Percentage Peak Area
1	Benzyl benzoate	1787	0.04
2	Octadecane	1797	0.02
3	Nonadecane	1897	0.09
4	Hexadecenoic acid	1982	0.13
5	Eicosane	1997	0.06
6	Heneicosane	2101	1.11
7	Octadecanoic acid	2185	1.12
8	Docosane	2223	92.63
9	Tricosane	2299	1.2
10	Tetracosane	2396	0.08
11	Docosanal	2432	0.11
12	Data MS	2472	0.43
13	Pentacosane	2496	2
14	Diisooctyl phthalate	2532	0.88
15	Hexacosane	2595	0.1

**Table 3:** Flavonoid and anthocyanin profiles of white and dark pink *R. canina* determined by LC-MS.

Peak	Rt (min)	MW	Ion Mode		Compounds	Chemical Formula	Peak Intensity in White Flower	Peak Intensity in Dark Pink Flower	Maximum Absorbance	Mass Fragments
			[M + H]^+^	[M − H]^−^						
1	2.43	772	773	-	Quercetin-3-O-rutinoside-7-O-glucoside	C_33_H_40_O_21_	2.18 × 10^5^	4.26 × 10^4^	3.23 × 10^5^	343, 344, 361, 366, 193, 194
2	2.49	533	534	-	Pelargonidin 3-O-(6″-succinyl glucoside)	C_25_H_25_O_13_	1.15 × 10^5^	3.10 × 10^5^	1.34 × 10^5^	136, 343, 193
3	2.52	290	291	-	Epicatechin	C_15_H_14_O_6_	6.26 × 10^5^	9.81 × 10^5^	4.16 × 10^7^	136, 193, 209
4	2.67	534	-	533	Kaempferol 3-O-(6″-malonyl- glucoside)	C_24_H_22_O_14_	1.86 × 10^6^	1.27 × 10^6^	7.95 × 10^5^	180, 192, 226, 372
5	2.81	314	315	-	Dihydroxy-dimethoxy flavone	C_17_H_14_O_6_	4.84 × 10^5^	3.50 × 10^5^	1.43 × 10^5^	104, 116, 118, 183, 187
6	2.85	270	271	269	Apigenin	C_15_H_10_O_5_	4.41 × 10^7^	5.71 × 10^7^	4.03 × 10^5^	104, 116, 118
7	3.04	566	-	565	Quercetin-3-O-dipentoside	C_25_H_26_O_15_	8.90 × 10^4^	6.62 × 10^4^	9.69 × 10^4^	133
8	3.05	298	299	-	Apigenin-7,4′-dimethyl ether	C_17_H_14_O_5_	1.12 × 10^6^	7.26 × 10^5^	1.26 × 10^6^	174, 183, 329
9	3.9	170	-	169	Gallic acid	C_7_H_6_O_5_	8.57 × 10^66^	1.90 × 10^7^	1.19 × 10^7^	169, 171
10	4.06	788	-	787	Tetra-O-galloyl-hexoside		1.14 × 10^6^	3.12 × 10^6^	1.54 × 10^6^	169, 392, 393, 477, 786, 787
11	4.23	470	-	469	Valoneic acid dilactone	C_21_H_10_O_13_	1.03 × 10^7^	1.61 × 10^6^	7.47 × 10^5^	316, 317, 610, 611, 470, 484
12	4.64	318	-	317	Myricetin	C_15_H_10_O_8_	8.33 × 10^5^	9.23 × 10^6^	1.08 × 10^6^	316, 484, 485
13	4.70	434	435	-	Delphinidin-O-pentoside	C_29_H_33_O_18_	1.65 × 10^6^	2.39 × 10^6^	1.92 × 10^6^	612, 613, 435, 450, 288,
14	4.85	610	-	609	Rutin	C_27_H_30_O_16_	7.76 × 10^6^	1.15 × 10^6^	8.30 × 10^6^	609, 610, 612
15	4.89	448	449	-	Kaempferol-3-O glucoside	C_21_H_20_O	2.36 × 10^6^	1.96 × 10^6^	2.64 × 10^6^	612, 613, 392, 288, 289, 449,135, 245, 183
16	4.89	448	449	-	Cyanidin-O-hexoside	C_21_H_21_ClO_11_	2.36 × 10^6^	1.96 × 10^6^	2.64 × 10^6^	
17	4.89	448	449	-	Quercitrin	C_21_H_20_O_11_	2.36 × 10^6^	1.96 × 10^6^		
18	5.89	302	303	-	Delphinidin	C_15_H_11_O_7_	1.97 × 10^7^	1.38 × 10^7^	2.23 × 10^7^	303, 286, 142, 205, 164
19	5.92	286	287	-	Cyanidin	C_15_H_11_O_6_	1.15 × 10^6^	1.06 × 10^6^	1.37 × 10^6^	303, 286, 304, 205, 164
20	6.21	302	-	301	Ellagic acid	C_14_H_6_O_8_	7.04 × 10^5^	2.87 × 10^6^	7.68 × 10^5^	301, 302, 603, 604
21	7.17	302	-	301	Quercetin	C_15_H_10_O_7_	1.60 × 10^6^	4.32 × 10^5^	7.68 × 10^5^	
22	7.68	600	-	599	Flavonol diglycoside		1.26 × 10^7^	1.14 × 10^7^	1.63 × 10^6^	599, 600, 601
23	7.74	464	465	-	Quercetin-3-O-hexoside	C_21_H_20_O_12_	1.32 × 10^6^	1.76 × 10^6^	1.54 × 10^6^	288, 289, 450, 464, 465, 288, 289
24	7.94	448	-	447	Kaempferol-3-O-glucoside	C_21_H_20_O_11_	3.73 × 10^6^	5.04 × 10^6^	4.20 × 10^6^	447, 448
25	8.87	418	-	417	Kaempferol-3-O-pentoside	-	1.20 × 10^6^	1.37 × 10^6^	2.68 × 10^5^	416, 417, 625, 626
26	9.90	594	-	593	Kaempferol-3-O-rutinoside	C_27_H_30_O_15_	8.47 × 10^5^	2.60 × 10^6^	3.83 × 10^5^	449, 450, 593, 594, 595, 596
27	9.90	594	-	593	Kaempferol-3,7-hexose-rhamnoside	-	8.47 × 10^5^	2.60 × 10^6^	3.83 × 10^5^	
28	11.85	286	-	285	Kaempferol	C_15_H10O6	1.14 × 10^6^	3.49 × 10^5^	1.61 × 10^5^	190, 192, 372
29	12.41	290	-	289	Catchin	C_15_H_14_O_6_	2.44 × 10^6^	7.35 × 10^6^	3.98 × 10^5^	288, 289
30	14.82	592	593	-	Cyanidin 3-O-(6″-dioxalyl-glucoside)	C_25_H_20_O_17_	8.26 × 10^5^	7.06 × 10^4^	1.12 × 10^5^	225, 236, 237, 360, 519, 521
31	14.91	432	433	-	Cyanidin-O-deoxyhexoside	-	1.16 × 10^5^	4.29 × 10^5^	1.63 × 10^5^	236, 237, 330, 446
32	14.91	432	433	-	Apigenin-5-O-glucoside	C_21_H_20_O_10_	1.16 × 10^5^	4.29 × 10^5^	1.63 × 10^5^	236, 237, 330, 446
33	15.06	616	617	-	Cyanidin 3-O-sambubioside	C_26_H_29_ClO_15_	6.10 × 10^5^	3.66 × 10^5^	2.79 × 10^5^	236, 618, 330, 360
34	15.31	610	611	-	Cyanidin 3,5-O-diglucoside	C_27_H_31_O_16_Cl	2.52 × 10^5^	2.25 × 10^5^	1.24 × 10^5^	142, 183, 225, 230, 236

**Table 4:** Primer sequences used in qRT-PCR for gene expression analysis.

Target Gene	Gene Bank Accession Number	Primer Sequences (Sequence in 5′-3′ Direction)
*PAL*	MG922976.1	F: GAGTACAGGAAGCCAGTGGT R: CCATAGCTGTCCGTACCCTT
*FLS*	AB038247.1	F: AAGGGTGGGTGGATCATCTG R: CATCACCACCAACTGCCTTC
*CER1*	XM_024319816.2	F: GGGAGATGGGTTGGTCATGA R: CGATCAACAGAGTTGCCACC
*AAT1*	MG820126.1	F: GCCCTCACTGGTTTTCTCTG R: GCTCCCTGGTGCTGTATCAT
*MYB1*	EU082130.1	F: CTATGTCAAGACTCGCACGC R: CAACGAGTGCAGGTGAGATG
*GGPPS*	KX661005.1	F: CACAAAACTGCGGCTCTTCT R: AGTCCTTCCCAGCAGTCTTC
*PAR*	AB426519	F: ACAGACCCAAAGGCAGAAC R: TCATCAACCACTACATCAGGAG
*ANS*	BI977949	F: GCTCGTCAACAAGGAGAAGG R: GGTAGAGG CGAGAGCTTCCT
*CCD1*	EU327776.1	F: CGAAAATTGAGGTTGGAGGA R: GCATGGAA CCCATATGGAAC
*DXR*	JX518618.1	F: GTGACCTCACCTTCCCTC R: CTACGCCACATCTACCAG
*GPS*	DQ286930	F: TGGCAACTGTTGTGGAACAT R: AGCACGAGACTTCAGCACT
*LIS1*	AGB14629	F: ATGGCTGAGTGTGAGTGTGA R: AGCTTTTGTTTATGGCCGGC
*DXS*	ACD70396	F: GGGTTACCTTGATTCCGACA R: CAACTTTTGCTGCCAGTTCA
*beta-actin*	RXHM01003052.1	F: GGGGAAAATATGGCATCACACG R: GATTGCGACATACATTGCTGGG

## Data Availability

The data presented in this study are available on request from the corresponding author.
